# Neurodegeneration exposes firing rate dependent effects on oscillation dynamics in computational neural networks

**DOI:** 10.1371/journal.pone.0234749

**Published:** 2020-09-23

**Authors:** David Gabrieli, Samantha N. Schumm, Nicholas F. Vigilante, Brandon Parvesse, David F. Meaney

**Affiliations:** 1 Department of Bioengineering, University of Pennsylvania, Philadelphia, Pennsylvania, United States of America; 2 Department of Neurosurgery, University of Pennsylvania, Philadelphia, Pennsylvania, United States of America; University of Modena and Reggio Emilia, ITALY

## Abstract

Traumatic brain injury (TBI) can lead to neurodegeneration in the injured circuitry, either through primary structural damage to the neuron or secondary effects that disrupt key cellular processes. Moreover, traumatic injuries can preferentially impact subpopulations of neurons, but the functional network effects of these targeted degeneration profiles remain unclear. Although isolating the consequences of complex injury dynamics and long-term recovery of the circuit can be difficult to control experimentally, computational networks can be a powerful tool to analyze the consequences of injury. Here, we use the Izhikevich spiking neuron model to create networks representative of cortical tissue. After an initial settling period with spike-timing-dependent plasticity (STDP), networks developed rhythmic oscillations similar to those seen *in vivo*. As neurons were sequentially removed from the network, population activity rate and oscillation dynamics were significantly reduced. In a successive period of network restructuring with STDP, network activity levels returned to baseline for some injury levels and oscillation dynamics significantly improved. We next explored the role that specific neurons have in the creation and termination of oscillation dynamics. We determined that oscillations initiate from activation of low firing rate neurons with limited structural inputs. To terminate oscillations, high activity excitatory neurons with strong input connectivity activate downstream inhibitory circuitry. Finally, we confirm the excitatory neuron population role through targeted neurodegeneration. These results suggest targeted neurodegeneration can play a key role in the oscillation dynamics after injury.

## Introduction

Traumatic Brain Injury (TBI) is a prominent cause of disability in the US [1]. Perhaps due to growing awareness of the consequences of TBI, emergency department visits for TBI increased 47% from 2007 to 2013 [2]. Although many of these injuries produce no long-term deficits, a fraction of injuries produces cognitive and psychological impairments that can last years after the original insult [3–5]. As a result, more than 5 million people in the US live with significant consequences of TBI, contributing to an estimated 70 billion dollars annually in medical and non-medical costs [6]. Effective recovery from TBI remains a challenge because no two injuries are exactly alike, leading to a unique injury and recovery pattern for each TBI patient.

One key feature in TBI recovery is how the structural and functional networks in the brain evolve over time after injury to guide the cognitive recovery processes [7–11]. Recent studies have shown alterations in brain circuitry after moderate and severe injury affect the coordination among functional brain networks [12]. With the development of models to predict the overall changes in brain networks during different tasks, there is an emerging consensus that the dynamic network that connects different brain regions can influence cognitive and psychological alterations after injury [13–16]. However, the unique circumstances that cause each TBI make it difficult to predict which injuries will likely lead to long-term changes in brain function. These challenges exist especially at the cellular scale, where the neuronal degeneration that may occur days to weeks after a TBI can alter the function of local microcircuits throughout the brain [17, 18].

To this end, computational models can be a useful tool to understand how neuronal damage ultimately contributes to the impairments in circuit function after TBI. In general, these models can account for the mechanisms of acute injury to the network (e.g., primary axotomy, membrane permeability changes, receptor dysfunction) and secondary changes that can also trigger neuronal loss [18–22]. Despite the many experimental methods to explore neural activity at different scales (e.g., single unit recording, local field potential recordings representing the aggregate activity of neuronal ensembles, and high speed calcium imaging to explore neuronal activation in awake animals), it is challenging to develop a precise relationship between neurodegeneration and network dynamics with these techniques [23–26]. Furthermore, models are adept at manipulating network features which are less accessible experimentally. For instance, the percentage of inhibitory neurons is known to vary depending on the specific neural circuit [27, 28] and is a feature of interest in this work. Finally, computational models can systematically examine the effect of damaging neurons within an integrated network without the influence of variable upstream circuitry. We can gain critical information that would be impossible or impractical to acquire using conventional methods.

Building upon past studies that examined how neuronal connectivity and injury patterns can lead to activity patterns which resemble posttraumatic epilepsy [29, 30], we use a computational model to examine the effect of neurodegeneration on the spontaneous activity of neural circuits. We utilize microcircuits that resemble isolated cortical circuitry to identify the exact relationship between local changes in network function and degeneration without the complexity of large-scale interconnected topology. We focused our work on how rhythmic oscillations developed in our networks, how spike-timing-dependent plasticity enhanced the recovery of these circuits after degeneration, and how degeneration in populations of neurons can play specific roles in altered network function. Together, our results demonstrate how neurodegeneration affects the dynamics of a microcircuit and the importance of spike-timing-dependent plasticity in repairing damaged microcircuits after injury.

## Methods

### Modeling a representative cortical circuit

**Fig 1:**
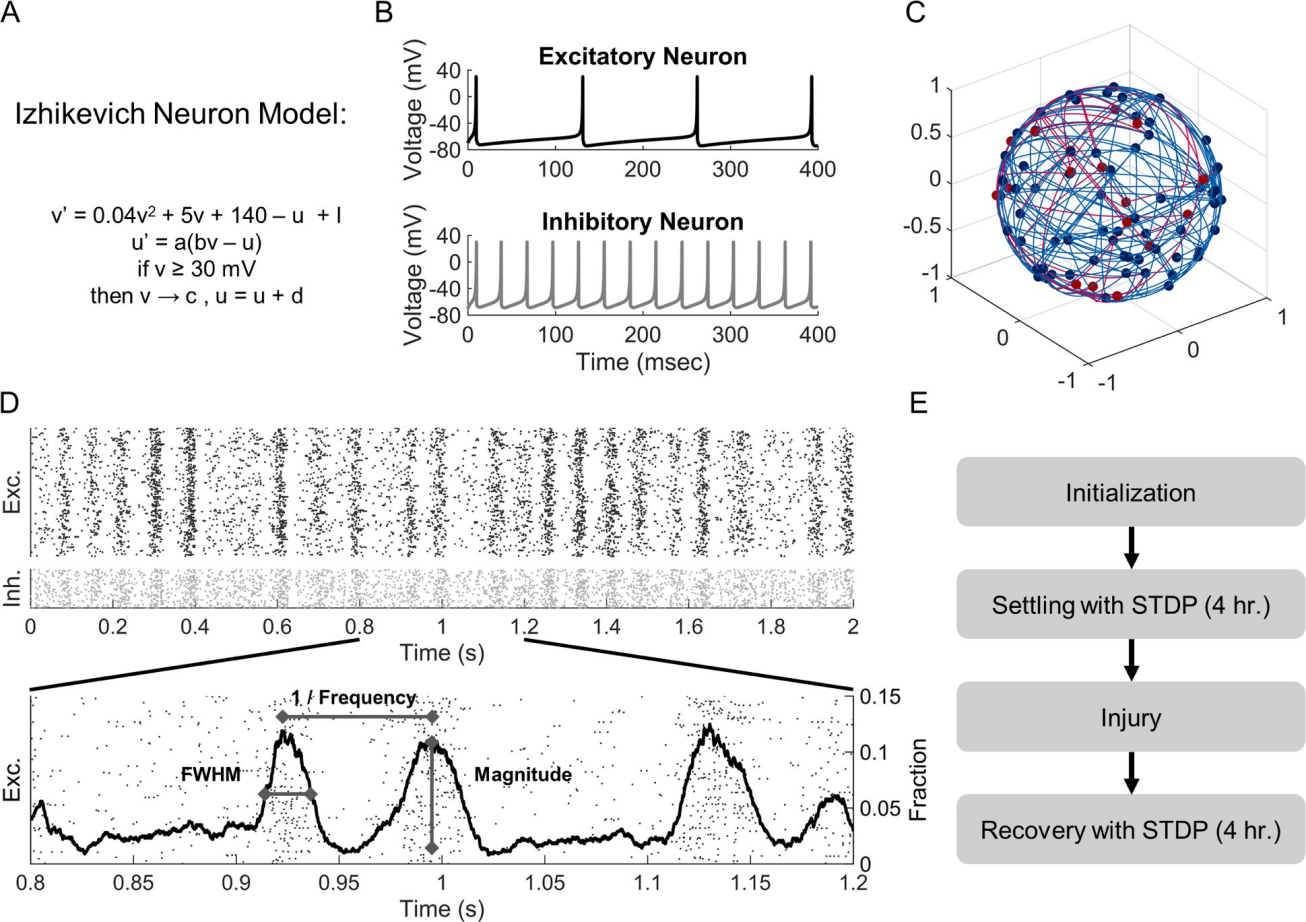
Schematic of methods. (A) The Izhikevich integrate-and-fire neuron model was used to simulate neuron activity. (B) Model parameters were tuned to represent regular-spiking excitatory (Exc.) neurons and fast-spiking, low-threshold inhibitory (Inh.) interneurons. (C) Neurons were randomly placed on the surface of a sphere and connected into a 1000 node network. (D) Networks developed rhythmic activity oscillations after a four-hour period of connectivity weight settling with spike-timing-dependent plasticity (STDP). (E) We characterized oscillations in our networks based on the number of oscillations per second, the peak number of spikes within the oscillation as a fraction of network size, and the full-width-half-maximum (FWHM). After network weights settled with STDP, we injured networks at damage levels from 5% to 95%. After recording activity metrics immediately after injury, the networks restructured connectivity weights, again according to STDP. We then reassessed network function.

To investigate the connection between degeneration and functional network activity, we constructed computational neural networks of integrate-and-fire neurons ([31]; Summary in Fig 1). Networks of 1000 neurons were constructed with 80% regular-spiking, excitatory neurons and 20% fast-spiking inhibitory to mimic the ratios commonly used to model cortical circuits [30–32]. While a ratio of 80% excitatory to 20% inhibitory neurons represents generic cortical tissue, we also explored the effects of excitatory/inhibitory balance on baseline network activity in an additional set of simulations. To do so, we created networks varying the percentage of excitatory neurons from 65% to 95%.

These neurons follow a system of ordinary differential equations to track membrane potential, membrane recovery, and threshold-based spiking as follows:

$$v^{\prime }=.04v^{2}+5v+140-u+I$$

$$u^{\prime }=a(bv-u)$$

$$if\;v\geq 30\;mV,then\;\left\{ \begin{array}{c} v=c\\ u=u+d \end{array}\right.$$

Where *v* represents the membrane potential in millivolts, and *u* is the membrane recovery variable. Parameters *a*, *b*, *c*, and *d* were set to create heterogeneous regular-spiking excitatory neurons and fast-spiking, low-threshold inhibitory neurons as in Izhikevich 2003 (Fig 1B). For each neuron, we allowed parameters *a*, *b*, *c*, and *d* to vary within a tight range to avoid network behavior resulting from a homogeneous neuron population. We used a fixed timestep (0.2 millisecond) and a forward Euler method to compute *v* and *u* over time.

Consistent with average firing rates *in vivo* and previous models using Izhikevich neurons [30, 33, 34], we used a gamma distribution (*k*, θ = 2, ½) to randomly (f = 1 Hz) inject currents into individual neurons within the network. This stimulation was strong enough to cause the neuron to fire and send AMPA- or GABA-based synaptic signals to downstream targets. Synaptic currents were modeled as exponential decays from AMPA or GABA_A_ receptors, with τ = 5 ms [32, 35–37]. Although there are other receptors with more slowly decaying currents, we focused on fast-decaying receptors to represent a large proportion of the synaptic current. AMPA and GABA receptors were calibrated to create excitatory or inhibitory post-synaptic potentials in accordance with past *in vivo* recordings [38]. Repeated input stimuli were attenuated at 40% immediately after a spike occurred (τ = 150 ms) to model desensitization in the neuron population.

To avoid bias from edge effects in seeding neuron position, we placed neurons randomly on the surface of a unit sphere ([30]; Fig 1C). The number of outputs for each neuron, which was drawn from a normal distribution with an average of 100 total outputs and inputs per neuron, varied slightly for each neuron to mimic features estimated in cortical circuits (10% variance; [39]). Neurons were randomly connected to each other across the surface of the sphere, producing network properties of a classic Erdos-Renyi random graph [40]. In a subset of simulations, we examined the effects of weak and strong distance-dependent connections and found that our main findings were unchanged. As a result, we present only the simulations using a random connection topology. Finally, we implemented synaptic transmission delays that were proportional to the distance between two neurons along the arclength of the sphere and set 8 ms as the maximum delay, consistent with *in vivo* recordings [41–45].

In neural networks, synaptic connection strengths adapt according to different models of synaptic plasticity. Among the models available, we chose to implement spike-timing-dependent plasticity (STDP) because of its critical role in learning and the potential role this feature may play in cognitive deficits after traumatic injury [46–48]. We used the Song model of STDP [49, 50], in which the synaptic strength was adjusted based on the relative timing of synaptic inputs to a neuron and the subsequent action potential firing of the target neuron. Mathematically, this can be described as:

$$\Delta w(w)=\left\{ \begin{array}{c} A_{+}(w)\exp\left(-\frac{t_{post}-t_{pre}}{\tau }\right)\;if\;t_{post}-t_{pre}>0\\ A_{-}(w)\exp\left(-\frac{t_{post}-t_{pre}}{\tau }\right)\;if\;t_{post}-t_{pre}\leq 0 \end{array}\right.$$

Where *w* is the synaptic weight of the connection between the pre- and postsynaptic neuron; *A*_*+*_ and *A*_*-*_ determine the maximum synaptic modification; *t*_*pre*_ and *t*_*post*_ are the timing of the pre- and postsynaptic activations; and *τ* is the plasticity time constant of 20 ms. Importantly, STDP was implemented at excitatory-excitatory synapses only. While there is evidence for STDP at other synapses (e.g., excitatory-inhibitory), inhibitory plasticity is complex and variable, depending on features like cell type, dendritic location, and neuromodulation [47, 51]. Until there is more consensus around these alternative forms of STDP, we and others have focused on well-characterized excitatory-excitatory synapses for computational modeling. Given that this formulation of STDP leads to a bimodal distribution of synaptic weights, in which weights approach either the minimum or maximum possible weight [49], we seeded the synaptic strengths in our networks using a bimodal distribution. For inhibitory neurons, which do not have STDP in the model, we used a normal distribution of synaptic weights 10% variance. We scaled the synaptic weights of excitatory and inhibitory connections from the starting distributions [0 1] to [0 4] for excitatory neurons and to [–14 0] for inhibitory neurons. These scales were chosen to correspond to excitatory and inhibitory postsynaptic potentials recorded *in vivo* [38].

For each network, we constructed the network topology and assigned synaptic weights between connected neurons. We then allowed the network architecture to reweight with STDP until the firing behavior of neurons achieved activity that did not vary in firing rate or average oscillation rate by more than 1% over a 5-minute simulation period. Across a range of connection architectures and synaptic weights, we attained a stable activity pattern for simulation times of 4 hours. For each condition examined, we constructed and completed ten independent simulations, averaging the results from these simulations into a single group.

We recorded five measures from each simulation: the neuron activity rate, coefficient of variation of the inter-spike interval (CoV ISI), oscillation frequency, oscillation peak magnitude, and oscillation width. Activity rate was calculated as the average firing rate of neurons within the network over a five-minute period. To assess variability in this activity, we computed the CoV (standard deviation over the mean) of the intervals between spike times. The CoV ISI was computed for each neuron and averaged across the population. Higher values correspond to larger variation in spike timing. To evaluate the occurrence of oscillations, we recorded the number of action potentials occurring in a 10-millisecond sliding time window over the simulation time (Fig 1D). From these data, we selected times where peaks in activity occurred for the network (peak prominence ≥ 1) and used these times to compute the oscillation frequency as the number of oscillations per second. At each oscillation, we defined the oscillation magnitude as the maximum number of spikes within the sliding window and the width as the full width at half peak intensity.

We further analyzed each network oscillation to identify preferred spike timings for neurons. For each oscillation, we identified an interval that was 1.4 times the size of the oscillation width centered around the peak. We then split the interval into uniform deciles and assessed the likelihood for each neuron to fire within each time interval.

### Damaging the neural network

Random neuron injury: To mimic traumatic injury, neurons were randomly removed from the network after initial network reweighting with STDP. To maintain the initial excitatory/inhibitory balance of the circuit, 4 excitatory neurons were removed for every 1 inhibitory neuron. To assess the immediate effects of damage, we ran simulations without adjusting connectivity weights for 5 minutes, recording both the average firing rate and oscillation parameters over this time period. Next, we allowed networks to remodel with STDP with simulation times long enough to allow the firing rates and oscillation behavior to settle (4 hours). We then reassessed our metrics with a stable connectivity for an additional 5 minutes (Fig 1E).

Activity-based excitatory neuron injury: A second type of deletion scheme used the firing rate of individual neurons as the selection criterion for deletion. Once a given network restructured with STDP, we rank ordered the firing rate of each neuron within the network and deleted either the neurons with the lowest firing rate (LFR) or, alternatively, the neurons with the highest firing rate (HFR). Because the focus of this removal strategy was to determine the effects of activity-based deletion, we opted to only remove excitatory neurons. Similar to random neuron injury, we assessed the immediate effects of neuron removal with static connection weights for 5 minutes and then allowed the network to resettle for 4 hours.

### Statistical testing

To compare average activity and oscillation parameters between injury levels and baseline, we used one-way analysis of variance (ANOVA) with Tukey-Kramer post hoc test. Comparisons between injured and damaged networks at each injury level used t-tests with Bonferroni correction for multiple comparisons.

## Results

**Fig 2:**
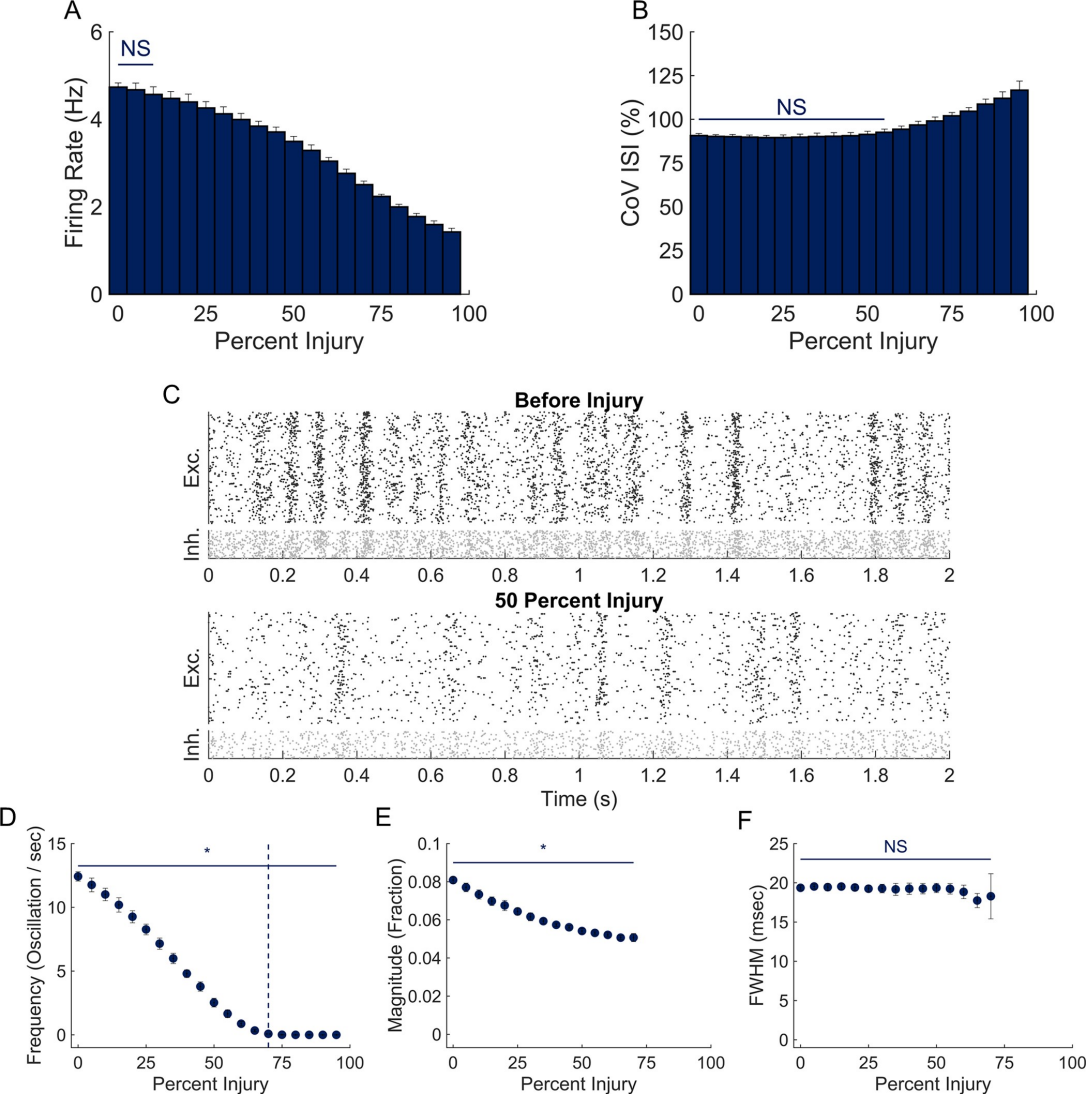
Effect of neurodegeneration on network dynamics. (A) Network firing rate was significantly reduced after 15% injury and continued to decline with further damage. (B) CoV ISI increased with higher levels of damage (60% and beyond). (C) Representative raster plots before and after 50% injury. (D,E) Network oscillation frequency and magnitude changed significantly from baseline at 5% injury. Oscillations were not consistently present in simulations with greater than 70% injury, demarked by the vertical dashed line. (F) Random neurodegeneration did not significantly impact oscillation FWHM.

To date, there is no clear evidence in the literature to suggest neurons with specific topological properties are more vulnerable than others to traumatic injury, although there are indications that specific regions of anatomic structures (e.g., CA3 in the hippocampus, cingulate, or thalamus) may preferentially show neuronal damage [52–54]. We first examined how the random deletion of neurons affected the pattern of neuronal activity in the networks. Without any neuronal deletions, networks had an average firing rate of 4.7 ± 0.1 Hz and an average oscillation frequency of 12.4 ± 0.4 Hz (Fig 2A, 2C and 2D). The CoV ISI was 0.91 ± 0.01 (Fig 2B). This activity level and the presence of oscillations are consistent with past computational models using similar methods [31, 32, 50].

Patterns of activity and oscillations slowly changed with the progressive deletion of neurons in the network. The average firing rate significantly decreased when deleting 15% or more of the network neurons (One-way ANOVA with Tukey Kramer post-hoc P < .001; Fig 2A). Concurrently, the CoV ISI remained stable until 60% removal at which point it increased, corresponding to increased variability in spike timing (Fig 2B). With significant changes appearing first in activity rate, the CoV ISI is interestingly more resilient to damage than the activity rate is. At the peaks in oscillation activity, we found that 8.1 ± 0.2% of the neuronal network was activated at baseline (Fig 2E). Random removal of neurons also led to a significant decrease in oscillation frequency and oscillation magnitude, with these changes appearing at lower damage levels (>5% or more deleted neurons; One-way ANOVA with Tukey Kramer post-hoc; p = 0.001 and p<0.001, respectively; Fig 2D and 2E). The duration of an oscillation was most resistant to neuronal loss, requiring at least 80% neuronal loss to show a significant decrease (One-way ANOVA with Tukey Kramer post-hoc; Fig 2F).

In a subset of simulations, we considered focal neuron removal, in which case we created a lesion by removing neurons that are physically near one another (S1 Fig). We found that the general trends were similar to those presented above and decided to proceed with random removal in subsequent studies. We attribute the similarity of these results to the random topology of our networks and anticipate that focal deletion might be more detrimental in a heavily distance-dependent network or another alternative topology. Furthermore, in a separate set of simulations, we investigated how altering the excitatory and inhibitory neuron subtype may alter general network dynamics after injury (S2 Fig). We find that our general results are similar for a variety of network compositions with differences only when chattering excitatory neurons were included.

**Fig 3:**
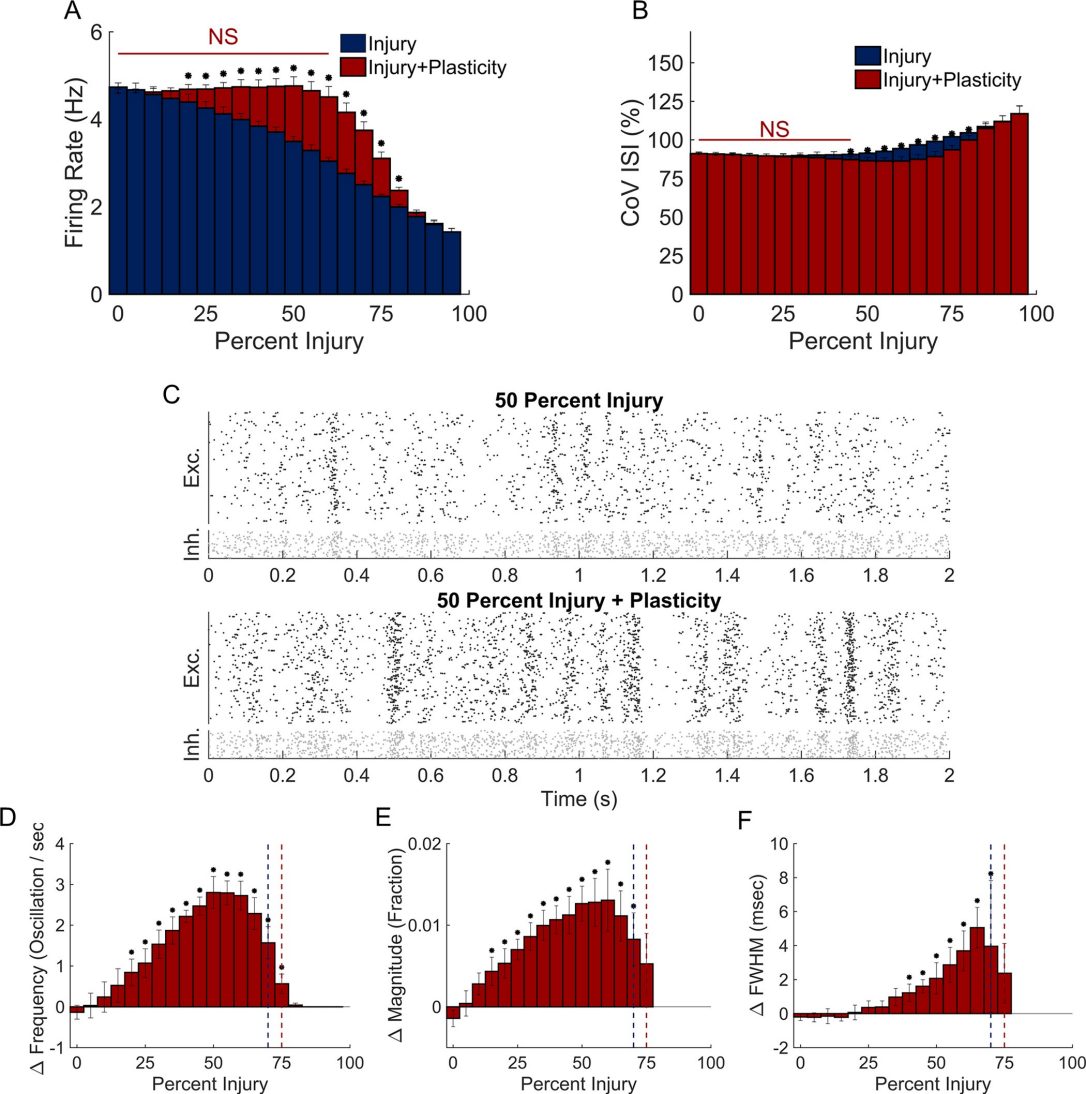
Role of plasticity in network recovery. (A) Four hours of recovery with STDP fully restored neuron activity back to baseline up to 60% damage. (B) With STDP, CoV ISI differed significantly from injury alone in the range of 45–80% damage. (C) Raster plots after 50% neurodegeneration and subsequent STDP recovery. (D,E) Network oscillation frequency and magnitude recovered significantly at moderate levels of damage (two sample t-test with Bonferroni correction for multiple comparisons). These changes were not sufficient to return to baseline, but partially restored function. Dotted vertical line at 70% and 75% injury denote the last injury that all simulations contained oscillations in pre- and post-plasticity simulations, respectively. (F) FWHM changes after plasticity were significantly different from baseline and significantly different from immediately post-injury in the range of 40–70% injury.

We next considered whether STDP would repair the functional deficits appearing in networks after damage. The average firing rate of neurons in the network increased significantly over a broad range of damage when STDP rebalanced synaptic weights (20–80%; Fig 3A; representative changes in Fig 3C). At lower levels of damage (5–60%), average neuronal activity was not significantly different from undamaged networks (Fig 3A). We also found that the CoV ISI did not differ from baseline at lower levels of removal (5–45%) and did significantly differ from networks without STDP in the range of 45–80% deletion (Fig 3B). Similar to firing rate, plasticity supported a significant increase in oscillation frequency and magnitude relative to networks in which the synaptic weight was held constant (Fig 3D and 3E). Unlike average firing rate, though, plasticity did not recover the oscillation frequency, magnitude, or duration to levels observed in undamaged networks. Beyond 25% damage, oscillation width significantly increased relative to undamaged networks (One-way ANOVA with Tukey Kramer post-hoc; Fig 3F).

**Fig 4:**
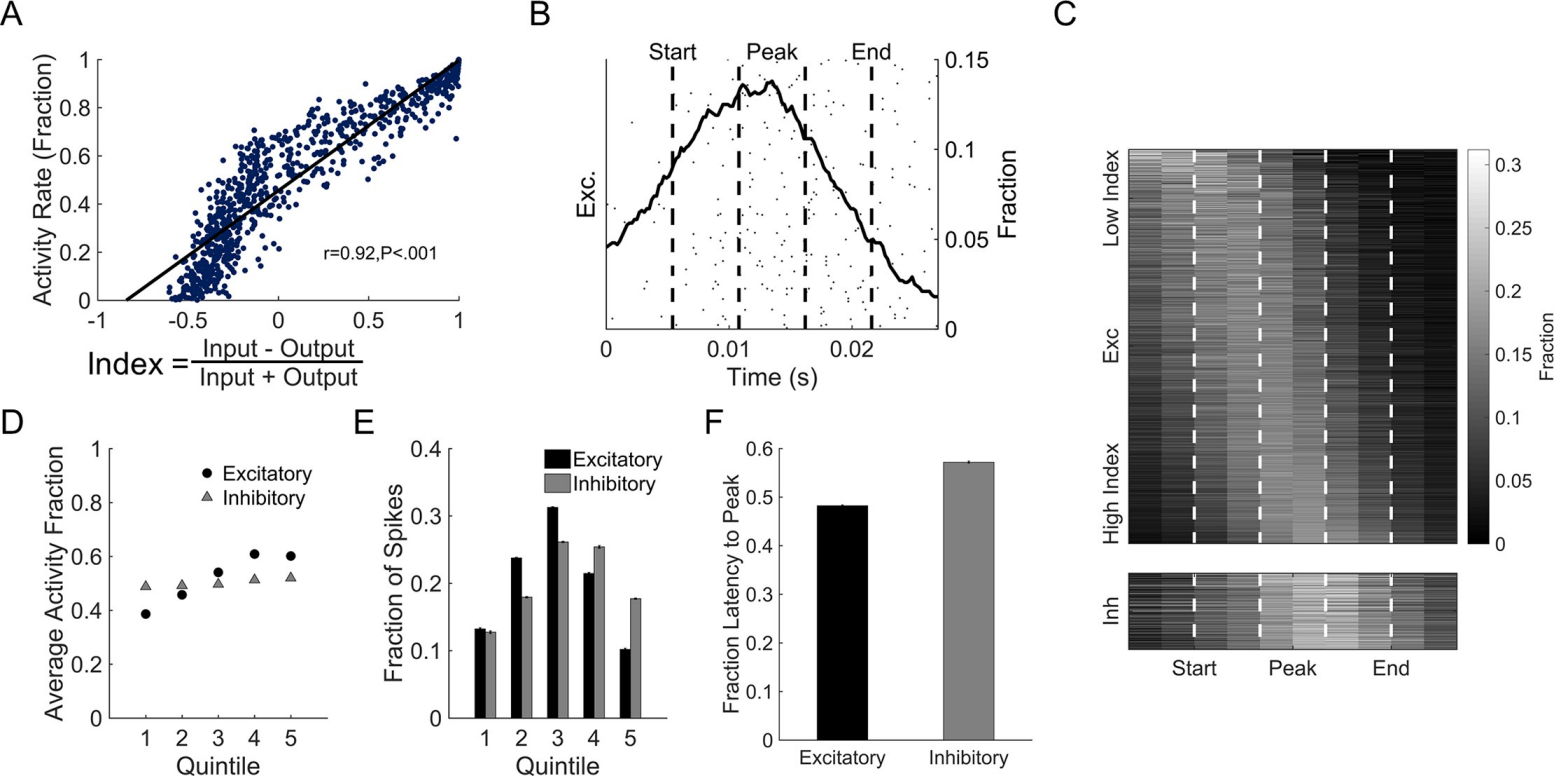
Activity-dependent functional roles of neurons. (A) After initial settling, networks developed structure-function relationships. We defined the neuron-connectivity index as the difference between input and output strengths normalized to total strength. Firing rate was significantly correlated with neuron-connectivity index. (B) To test the roles of neurons with different activity profiles in oscillations, we divided each oscillation into equal quintiles centered around the peak activation period. The labels and dashed lines correspond to those in panel C. We then tracked when each neuron fired within the oscillation. (C) Low index neurons tended to fire early in network oscillations, suggesting a role in oscillation initiation. High index neurons conversely fired at the peak of the oscillation and activated downstream inhibitory circuitry to stop oscillatory behavior. (D) Low activity excitatory neurons were more likely to be active early in oscillations. Later stages showed increased activation of highly active excitatory neurons. Inhibitory neurons showed no activity dependence in their firing time within oscillations. (E-F) Excitatory neurons had increased activation to peak oscillation magnitude, with decreasing activation after the peak. Inhibitory neurons had delayed activation, primarily firing at peak or late in the oscillation.

After observing that spike-timing-dependent plasticity introduced resilience to damage, we next explored if there were specific connectivity features of individual neurons that influenced or explained part of this resilience. For each neuron in an undamaged network, we computed a neuron connectivity index as the normalized difference of total synaptic input strength and the total output strength. This neuron-connectivity index correlated with the average neuronal firing rate; neurons with high index showed higher firing rates than neurons with low index did (Fig 4A). The relationship between input/output strength and firing rate was stable while the synaptic weights adjusted via STDP over 4 simulation hours. In addition, these relationships did not change across a broad range of neuronal network parameters that included synaptic strength, neuron parameters (a-d) that could change neuronal type [31], and connection number among neurons in the network.

We then sought to find if either the initiation or termination of oscillations was related to neuronal activity and, in turn, connectivity strength. Using our definition of the beginning and end of an oscillation (see Methods; representative oscillation appears in Fig 4B and 4C), we divided an oscillation period into quintiles. In general, excitatory neurons with low inputs relative to their outputs (i.e., low index) fired primarily during the initiation period of an oscillation, while high input strength excitatory neurons fired near the peak of an oscillation and activated inhibitory neurons to arrest the oscillation. The average firing rate of excitatory neurons within each of the first four quintiles significantly increased, while the firing rate significantly decreased in the fifth quintile (One-way ANOVA with Tukey Kramer post-hoc; p < .001; Fig 4D). In comparison, we could not identify any dependence on firing time and oscillation period for inhibitory neurons (Fig 4D). Excitatory neurons most commonly fired during the peak of the oscillation, decreasing on both sides of peak (Fig 4E). Inhibitory neurons had a preference to fire later in the oscillation, peaking between the 3^rd^ and 4^th^ quintile (Fig 4E). Together, these results indicate that the initiation of an oscillation corresponds with the activation of excitatory neurons with low firing rates, while the termination of the oscillation begins with the simultaneous recruitment of excitatory neurons with high firing rates and a significant fraction of the inhibitory neurons.

**Fig 5:**
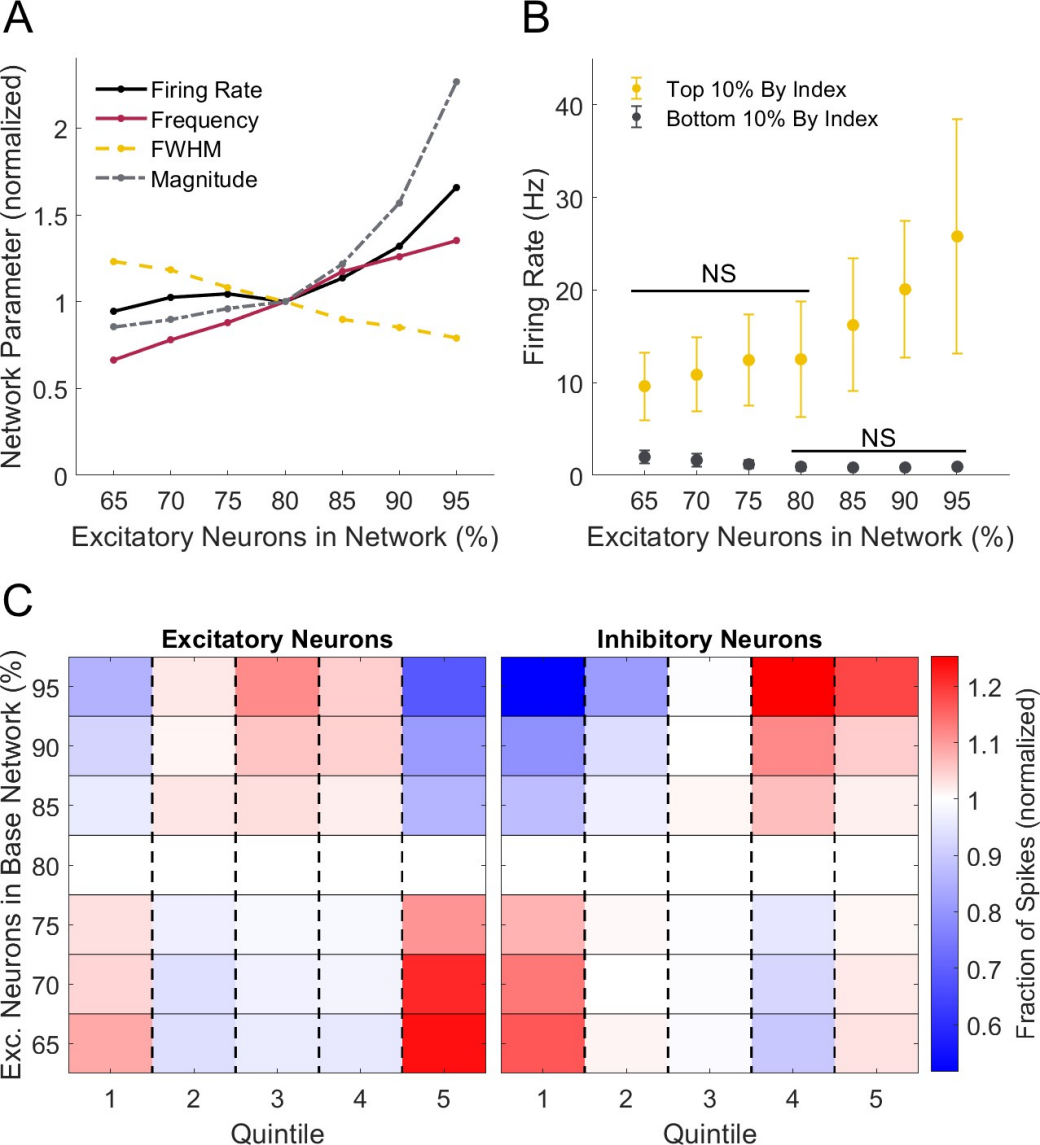
Changes in excitatory/inhibitory balance affect network dynamics. (A) Increasing the percentage of excitatory neurons in the network increased firing rate, oscillation frequency, and oscillation magnitude. In contrast, oscillation FWHM decreased with a higher proportion of excitatory neurons. All network parameters are normalized to the baseline (80% excitatory to 20% inhibitory) network. (B) The difference between average firing rate of neurons with the highest and lowest index values widened as the percentage of excitatory neurons increased. (C) As the percentage of excitatory neurons increased, the fraction of excitatory neuron spiking increased at oscillation peak and decreased at oscillation start and end, with most prominent changes occurring at the end of oscillations. With a lower percentage of excitatory neurons, opposite changes occurred with higher fractions of excitatory neuron spiking at the start and end of oscillations. As the proportion of excitatory neurons increased, the fraction of inhibitory neuron spiking decreased at start and increased at end of oscillation, with most prominent changes occurring at the start of oscillations. All spike fractions were normalized to the baseline (80% excitatory) network.

After observing differences in activation timing between excitatory and inhibitory neurons in the representative cortical circuit, we investigated how changing the excitatory/inhibitory (E/I) balance of the baseline network affected network activity, and in particular, when neurons spiked within each oscillation. To adjust the E/I composition of our networks, we seeded networks with a range of 65% to 95% excitatory neurons and compared these to the baseline network of 80% excitatory neurons. As expected for networks with more excitatory neurons, we found that the overall firing rate increased. With increasing proportion of excitatory neurons, we also found that the frequency of oscillations increased while the average oscillation width, or duration, decreased (Fig 5A). As the percentage of excitatory neurons in the network grew, the fraction of excitatory neuron spiking increased at the peak of oscillations, decreased at the beginning, and most sharply decreased at the end of oscillations (Fig 5C). Opposing shifts in the fraction of excitatory spiking were observed for networks with a low proportion of excitatory neurons (Fig 5C). Specifically, a larger fraction of spikes occurred at the beginning of the oscillation. For inhibitory neurons, increasing excitatory neuron composition in the network decreased the number of spikes before the oscillation peak and modestly increased it after the oscillation peak (Fig 5C). We found that the neuron-connectivity index (defined in Fig 4A) correlated with activity rate regardless of the E/I composition of the networks tested (Pearson’s correlation coefficient; r>0.7 and p<0.001 for all). The gap between the activity rates of the highest and lowest index neurons grew as the percentage of excitatory neurons in the network increased (Fig 5B). These results suggest networks comprised of more excitatory neurons exhibit shorter, more frequent oscillations, a likely result of two reductions in activity: excitatory neurons at the end of each oscillation (ending oscillations more quickly) and inhibitory neurons at the start of each oscillation (allowing oscillations to start more often).

**Fig 6:**
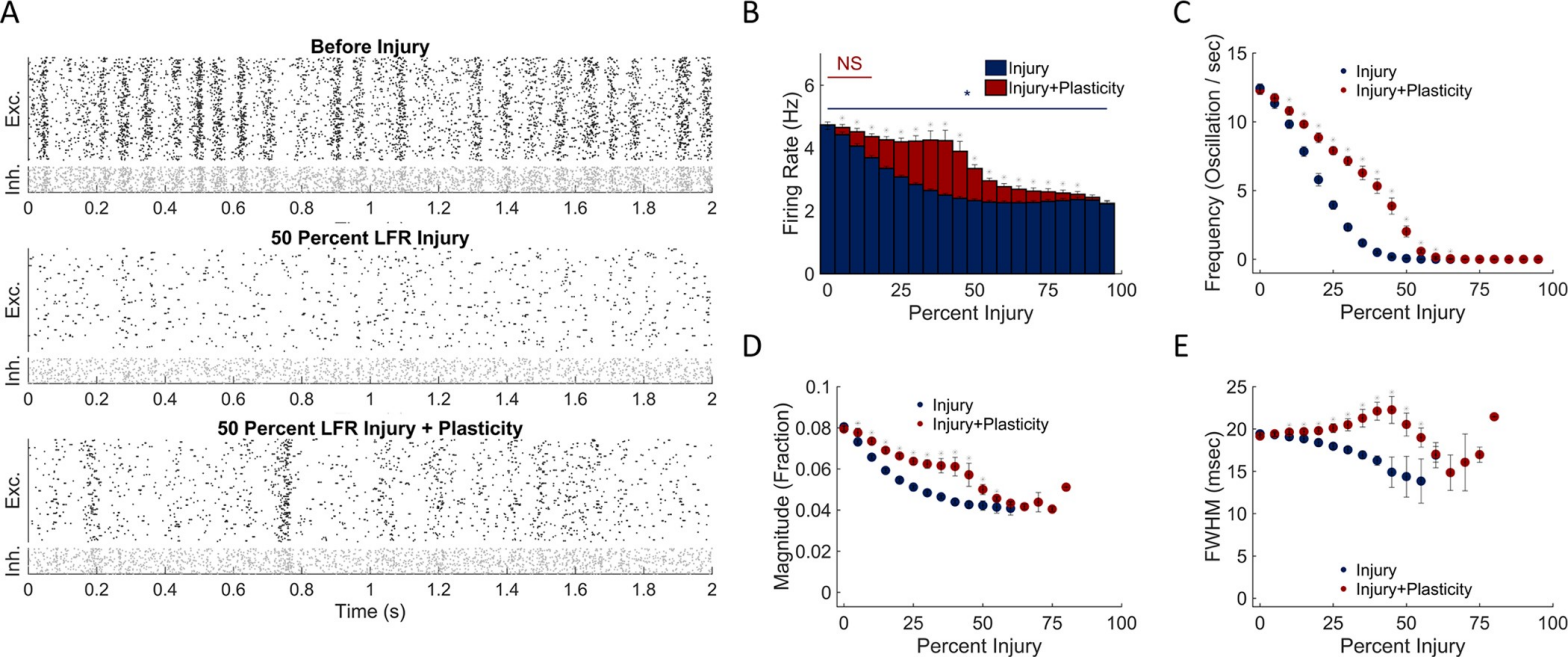
Effect of deleting relatively inactive neurons is partially recovered with plasticity. (A) Representative raster plots before injury, at 50% low firing rate (LFR) excitatory neuron injury, and after network restructuring with STDP. (B) LFR damage produced a rapid decline to baseline firing after injury that significantly recovered with STDP. (C,D) This restoration was primarily due to a recovery of network oscillation frequency and magnitude. (E) Changes in oscillation width occurred relative to baseline, primarily following damage exceeding the threshold of full firing rate restoration.

Since excitatory neurons with specific connectivity and activity patterns correlated with oscillation dynamics, we next considered if the targeted deletion of either activity type (low firing rate, LFR, or high firing rate, HFR) would alter the neural circuit dynamics. Using the random deletion of neurons as a comparison, we explored the change in functional network characteristics that would occur if we progressively deleted the neurons with the lowest average activity. With this strategy, we found that average activity rate would significantly decrease when damage exceeded 5% of the network (Fig 6A and 6B). Similar to random deletion, less damage was required to significantly change oscillation frequency (Fig 6C; damage > 5%) than activity rate. The deletion of the lowest firing rate neurons additionally led to significant change in the average width of an oscillation (Fig 6E). Plasticity returned the average activity in the network to baseline up to 15% damage; beyond this level, plasticity did improve average firing rate up to 90% injury (Fig 6A). In comparison, plasticity improved the oscillation frequency (and magnitude) over a range of injury levels (10–65% and 5–55% respectively), but it did not reach baseline levels.

**Fig 7:**
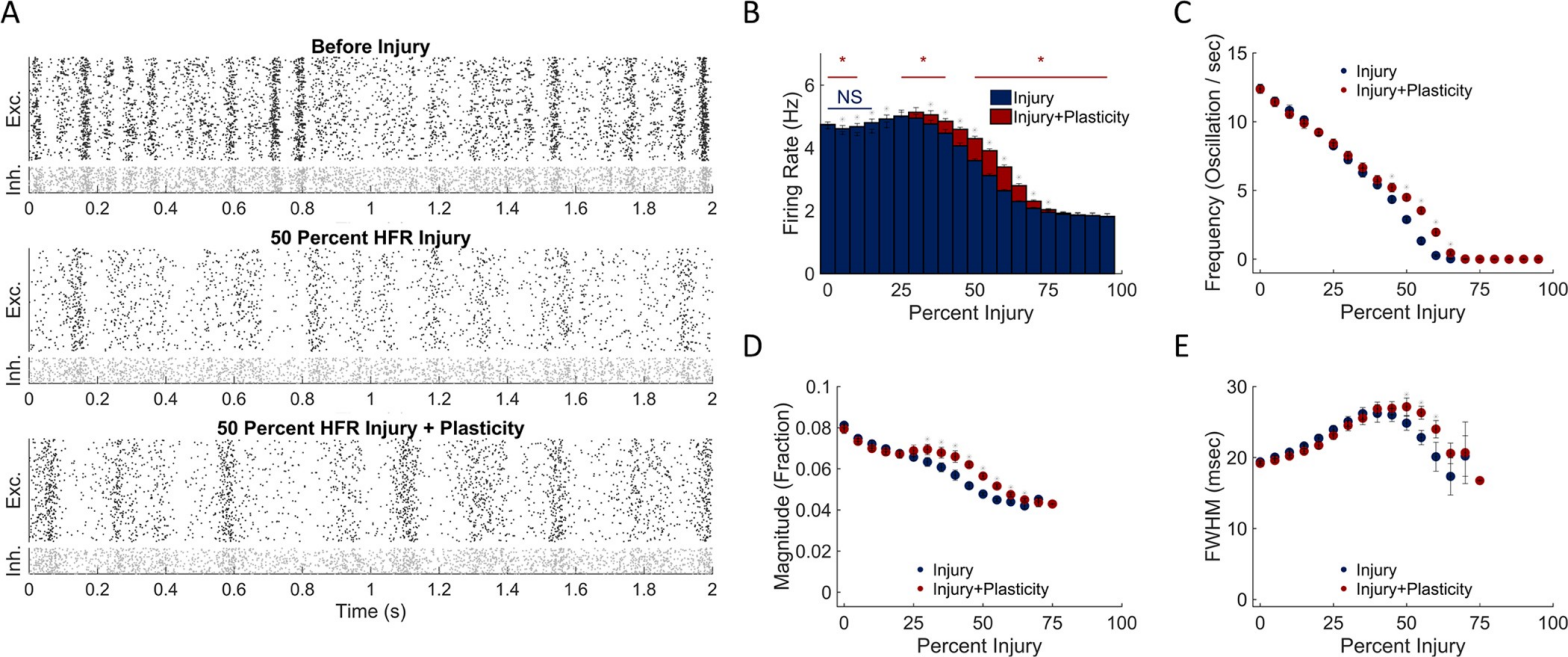
Removing highly active neurons predominantly alters oscillation dynamics. (A) Raster plots before injury, at 50% high firing rate (HFR) excitatory neuron injury, and after network restructuring with STDP. (B) Network activity did not significantly vary from baseline after HFR neurodegeneration. (C-D) Oscillation frequency and magnitude after plasticity were significantly different at the same injury level at higher levels of injury. (E) Oscillation width increased above baseline levels after damage and was maintained following restructuring with STDP.

In contrast to these results, the progressive deletion of neurons with the highest activity rate did not significantly change the overall average activity of the network until more than 20% of neurons were removed (Fig 7A and 7B). Removing the neurons with highest activity led to a progressive decrease in oscillation frequency that was significantly different from deleting the same fraction of low firing rate neurons (t-test with Bonferroni correction for multiple comparisons; p < .001; Figs 6C & 7C). Unlike the random deletion of neurons, the width of oscillations increased significantly over a broad injury range (15–55%; Fig 7E). While the oscillation frequency and/or magnitude changed with this injury approach, plasticity only modestly affected the oscillation frequency, magnitude, and average width at higher damage levels. (Fig 7C–7E).

To summarize the differences between deleting low vs. high firing rate neurons, removing low activity neurons produced nearly immediate changes in both activity and oscillation properties; however, these networks exhibited partial recovery to baseline after plasticity. Removing high activity neurons did not immediately reduce the network firing rate, but these networks did not show much recovery with STDP. Importantly, the mechanism of STDP is the same in both cases. The only difference was the subset of neurons which was removed, so any differences in recovery were due to the topological properties of those removed neurons. Our results show a greater potential for STDP-dependent recovery when LFR neurons were removed than when HFR neurons were removed.

## Discussion

Here, we modeled the effects of neurodegeneration on the functional dynamics of neural circuitry. We utilized an established spiking neural network model to investigate how damage and plasticity impact network firing and oscillatory behavior. In addition to establishing how unique connectivity patterns of neurons contribute to the development of oscillations, we show how those activity patterns are affected by E/I balance in the network. Further, we demonstrate that neurodegeneration, regardless of whether it was random or based on neuronal firing rate, significantly decreased network activity and oscillation dynamics. For all types of neurodegeneration, activity was significantly restored with spike-timing-dependent plasticity. Deleting highly active neurons led to a marked increase in oscillation duration, while deleting neurons with low activity reduced the initiation of oscillations without decreasing their frequency. These results suggest that the degeneration or inactivation of specific neuron activity profiles can differentially affect oscillation dynamics of neuronal circuits.

We used several simplifying assumptions to examine the potential impact of neurodegeneration. First, we use generalized topologies and neuron spiking behaviors of cortical neurons based on simplified rules developed from *in vivo* observations [30–34]. We recognize that neurons within specific brain regions can vary greatly in their connectivity preferences [55, 56], spiking behavior [57], and functional plasticity properties [51]; therefore, some of our observations on neuronal degeneration may not apply universally to all brain regions. We did explore whether our observations were influenced by different distance-dependent connection algorithms and found that the general decline in activity and the restorative effect of plasticity did not depend on the initial spatial connections in the network. We also appreciate that there have been documented forms of inhibitory STDP [51, 58–60] outside the classical excitatory-excitatory implementation we used in this work. It would be worth exploring the effect of these algorithms on synapses between more specific neuron models. Since inhibitory STDP characteristics vary considerably based on the cell type, it would be important to first identify a key brain region and cell types of interest. Although we anticipate that our general findings could inform predictions for networks of more complex, region-specific topologies, many of the regions commonly damaged in TBI (e.g., hippocampus, thalamus, and cingulate [52, 53]) lack clear estimates of neuronal connectivity. Once available, we envision creating specific computational network models to assess how dynamics differ with deletion across these specific regions.

Our second main limitation is that we used the Izhikevich integrate-and-fire neuron model to approximate neuron activity, which may not fully represent the complexity of *in vivo* circuitry. Furthermore, there are additional receptors beyond the fast-decaying AMPA and GABA_A_ receptors used to model synaptic activity here, and other slow-decaying receptors (e.g., GABA_B_, NMDA) have longer time constants [32, 36]. These additional receptors may alter action potential timing at the margins, but the parameters of the neuron model were chosen to accurately represent spiking behavior for these generic neurons given the currents we implemented. Since we were interested in evaluating network activity and oscillations, the most salient feature of our chosen neuron model was accurate spike timing. Because the Izhikevich model has been thoroughly validated on this feature for a variety of neuron spiking behaviors [31, 61–63], this model represents *in vivo* neural activity for the metrics we utilized in this study. Another neuron model would likely be a better choice for exploring detailed intracellular responses to injury. For instance, a multicompartment model would enable analysis of the dendritic response. Extending the current work into a more computationally complex neuron model with higher-order biological features (reviewed in [62]) could provide additional information into the timing of activation and synaptic inputs across an individual neuron [64]. However, these changes would likely affect all neurons in the network similarly and would not significantly impact the overall estimates of activity and our main findings. Finally, we considered whether the broad distribution of neuronal types employed in larger scale Izhikevich integrate-and-fire models were important to include in this study. Indeed, in a limited set of simulations, we found that excitatory neuron type did influence the response to mechanical trauma, implying that more complex circuits with more excitatory neuronal types may lead to more complex patterns of activity after injury. However, without first examining how more homogeneous circuits remodel in response to trauma, we believed this additional complexity would prevent one from gaining clear insight into which types of neuronal deletion would be particularly damaging to circuit dynamics.

In general, our neural activity patterns match the general spiking patterns and network-wide oscillations in both computational networks of similar size (e.g., [29, 31]) and more complex networks of larger size [32]. One key characteristic we observed in our simulations was the coordinated activation of a subset of neurons in regular periodic intervals over the entire simulation period. Again, these waves of neuronal activation are distinct from a near simultaneous activation or bursting of the network that can appear in some studies [29]. These oscillations of neuronal activity, where 10–15% of the network was activated, were the first to show a significant decrease in oscillation frequency after neurons were deleted from the network. Although neuron characterization traditionally depends on functional spiking properties of neurons [65–67], our results show that plasticity is an important mechanism to produce functional diversity. Moreover, our results identified subpopulations of neurons that could either trigger or suppress these periods of high network activity. To our knowledge, showing that neurons with low activity rates preferentially activate oscillations in a network has not been reported in past modeling studies, nor are we aware of past reports showing that neurons with high firing rates are important for quieting periods of high activity. Together, these data suggested that targeting specific neuronal populations for degeneration would preferentially affect neuronal dynamics, a prediction we confirmed with subsequent simulations.

The rhythmic oscillations may also have important consequences on the synchronization, or coherence, of activity across brain regions. Coherence among neuron populations is important for attention and memory [68–70], cognitive processes that are commonly affected after traumatic brain injury. Selective degeneration of low firing rate neurons reduces the likelihood of initiating an oscillation, in turn lowering coherence with other brain regions downstream of the injured microcircuit. Therefore, losing this neuronal subpopulation would appear to play a significant role in information relay across brain regions, an aspect of information processing that has appeared in network-based studies of TBI [13, 71]. In comparison, losing neurons with high firing rates would appear to have less consequence on the coordinated oscillations among regions because these neurons do not affect the emergence of an oscillation and only slightly lengthen the oscillation duration. However, we cannot completely discount the impact of losing high firing rate neurons because lengthening a specific oscillation may impede the propagation of sequential information across nodes in a network. Together, these point to the possibility for a small number of neurons to play a large role in relaying information, via oscillations, across several interconnected microcircuits.

Our general finding that spike-timing-dependent plasticity (STDP) is a key mechanism to re-stabilize network dynamics provides a potentially new role for STDP in the injured brain. As a primary mechanism associated with Hebbian learning, STDP is typically considered as a mechanism to restructure the synaptic connections in a network after a training stimulus [72, 73]. The return of activity to a damaged network using STDP is reminiscent of how homeostatic plasticity allows a healthy network to gravitate towards a target activity rate [74]. Interestingly, homeostatic plasticity may play a very different and destabilizing role in networks after either focal or more diffuse deafferentation of neurons [29], leading these networks into brief bursts of activity that resemble interictal discharges that appear in posttraumatic epilepsy [75, 76]. However, this rebalancing of networks with STDP has its limits, as the relative success of recovering initial dynamics is not complete at the highest injury levels. In light of several reports showing that one form of plasticity–long-term potentiation (LTP)–is lost after traumatic injury *in vivo* and *in vitro* [48, 77], our results emphasize the importance of therapeutic strategies to help promote plasticity after injury and regain initial network dynamics. Similarly, given that oscillations can be important to establish coherence among brain regions, steps to maintain plasticity in an injured network would likely improve network communications in the traumatically injured brain.

Our results also provide some suggestions on how local damage in the brain may affect both local and global brain dynamics. Cognitive disruptions from TBI are frequently viewed as changes in the network structure among regions in the brain (reviewed in [13, 14, 71, 78, 79]). Commonly, past studies focus on the functional and structural deficits in the connections among nodes in the network resulting from diffuse axonal injury [52, 71, 80]. With new techniques in medical imaging and network theory, we can begin to understand how changes in the connectivity between regions can impact higher level cognitive function [81–83]. However, these approaches rely on maintaining function at the node (microcircuit) level after injury. Certainly, we know there are regions of the brain that can impart large cognitive deficits simply with their own malfunction [71, 84]. From the current work, we know that neurodegeneration can impact both network activity and neural oscillations in the node. In combination, our results suggest that damage to one node (microcircuit) could indirectly influence the coordination of activity across many connected brain areas.

One method by which local damage could impact coordinated neural activity across a broad network is through shifts in the balance of excitation and inhibition. Specifically, our work shows that networks with high excitatory tone developed shorter, more frequent oscillations than their low excitatory tone counterparts did. This result is supported by the existing literature and carries important implications in the context of traumatic brain injury. Previous work indicates the importance of GABAergic inhibition for developing oscillatory rhythms in cortical circuits [85]. Other studies established that TBI alters E/I balance across different brain regions as well as via various injury modalities and severities [86–90]. E/I balance could shift due to selective vulnerability of interneuron subpopulations or due to changes in excitatory synaptic transmission. Regardless of the mechanism, our results suggest that changes in E/I balance post-injury modify the pattern of information generated by that local circuit. This would transform not only the local signaling within that sub-circuit but also the way the region communicates with other sub-circuits [91]. More study is required to determine the precise injury and recovery trajectory of networks with selectively removed inhibitory subpopulations. Inhibitory tone also is known to vary depending on the brain region as well as species. For example, in the thalamus alone, the percentage of interneurons varies from 4 to 16% in different nuclei of the murine thalamus, with reported values up to 30% in other species [28]. Based on our analysis of networks with varying percentages of inhibitory neurons, it is important to accurately represent the balance of excitation and inhibition in the network when assessing the impact of neurodegeneration on a specific circuit. Given the importance of the hippocampus in memory and TBI, our lab is working to further study this question within a network model of the hippocampal circuitry.

It is certainly plausible to explore some of the unique consequences of local neurodegeneration in broader brain networks using oscillator or neural mass models to link structural and functional networks of the brain [92, 93]. Although these models capture gross behavior of network dynamics, the current formulation of these models lacks the nodal accuracy to determine how perturbations caused by injury can impact the larger network function. Similar to work showing how local gamma activity could create biologically realistic BOLD correlations [32], changes in network oscillation frequency or oscillation width would likely have far-reaching impact beyond the local network. Particularly important would be examining the implications of an inconsistent oscillatory rate, as seen in our model, in a connected oscillatory model. To our knowledge, this feature is not commonly explored in neural mass or oscillator-based models. There is evidence to suggest that these models have synchronization properties that are sensitive to nodal dynamic changes, but oscillators with individually variable frequencies have yet to be investigated [94, 95]. Similarly, both neural mass and oscillator-based models can potentially benefit from further analysis of biological plasticity mechanisms to repair damage, given the role we found for plasticity in stabilizing or practically recovering nodal dynamics.

Overall, this study indicates that neurodegeneration alters population-level activity and network oscillations, with subpopulation-dependent changes to oscillation frequency or duration. These changes in network dynamics can be significantly recovered with spike-timing-dependent plasticity. We anticipate that future work in brain network dynamics will develop insight to discriminate between specific patterns of damage that cause long-lasting alterations in brain dynamics and other patterns of damage that produce temporary changes in neural dynamics. At a higher level, distinguishing between these two injury patterns can help identify injuries that could cause lasting cognitive deficits much earlier than currently possible, pointing to an opportunity to treat and improve outcome in a vulnerable population of TBI survivors.

## Supporting information

S1 Fig Effect of focal neurodegeneration on network dynamics. (A) Network firing rate decreased significantly after 35% injury and continued to decline with further damage. (B) CoV ISI increased with higher levels of damage (60% and beyond). (C,D) Network oscillation frequency and magnitude changed significantly from baseline at 5% injury. Oscillations were not present in simulations with greater than 80% injury, marked by the vertical dashed line. (E) Random neurodegeneration did not significantly impact oscillation FWHM until 60% injury.
(PDF)

S2 Fig Electrophysiology and spiking behaviors of neurons affect network response to damage. (A) Networks consisting of RS or IB excitatory neurons had reduced average network firing rate compared to uninjured baseline networks at 25% damage. Networks with CH excitatory neurons responded less to injury and, thus, sustained activity near baseline levels. (B) At 50% damage, networks with RS or IB excitatory neurons showed larger decreases in firing rate. Networks with chattering neurons remained resilient. Inhibitory neuron subtype did not significantly affect the network response to damage.
(PDF)
