# Supplementary material for: Neurodegeneration exposes firing rate dependent effects on oscillation dynamics in computational neural networks
Source: PLoS One. 2020 Sep 23;15(9):e0234749. doi: 10.1371/journal.pone.0234749 (PMC7510994; doi:10.1371/journal.pone.0234749)
Supplement: S1 Fig — (A) Network firing rate decreased significantly after 35% injury and continued to decline with further damage. (B) CoV ISI increased with higher levels of damage (60% and beyond). (C,D) Network oscillation frequency and magnitude changed significantly from baseline at 5% injury. Oscillations were not present in simulations with greater than 80% injury, marked by the vertical dashed line. (E) Random neurodegeneration did not significantly impact oscillation FWHM until 60% injury. (PDF) [file pone.0234749.s001.pdf]

S1 Fig Supplementary Information

In a subset of simulations, we compared truly random deletion (as presented in the manuscript) with focal deletion. For this focal approach, we selected a random starting point and removed neurons radiating outward from that physical location. There were some differences from the simulations shown in Fig 2 of the manuscript, but the general trends of our findings are overwhelmingly similar. The similarity of the results is attributable to the random topology of the networks in both cases. As a result, neurons that are physically near one another may have connectivity features that are as distinct as neurons that are physically far from each other. The differences we do observe arise primarily because the propagation delays between removed neurons are smaller in the case of focal removal. We anticipate the differences between random and focal deletion would be greater in distance-dependent or other network topologies.

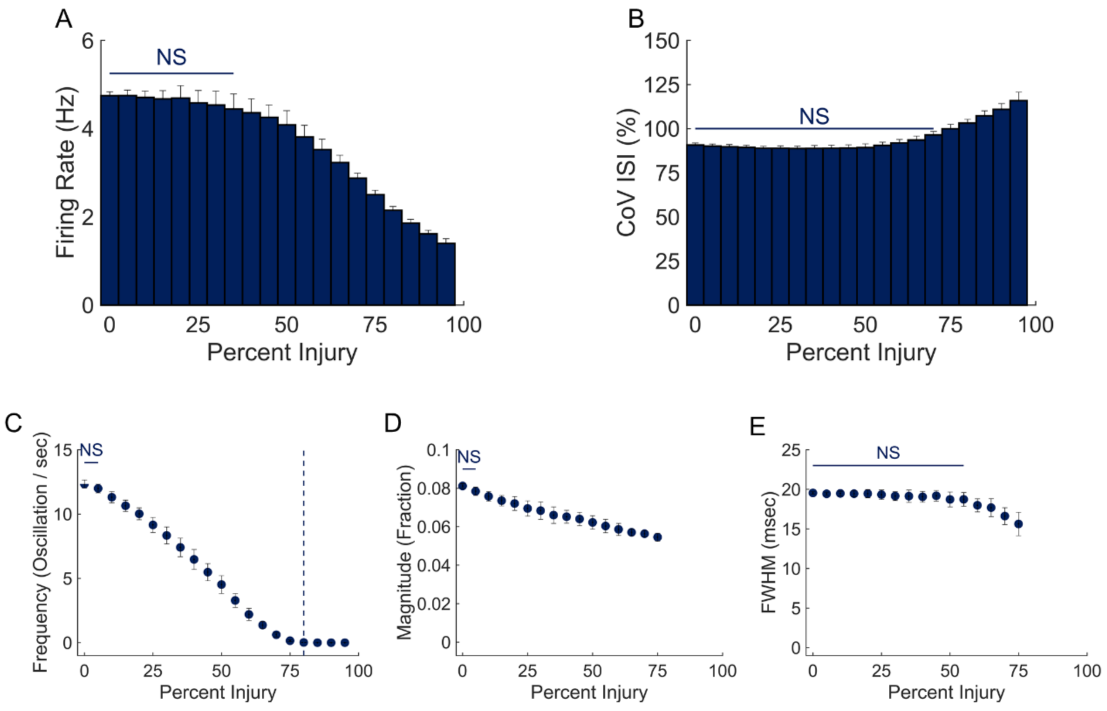

S1 Fig. Effect of Focal Neurodegeneration on Network Dynamics. (A) Network firing rate decreased significantly after 35% injury and continued to decline with further damage. (B) CoV ISI increased with higher levels of damage (60% and beyond). (C,D) Network oscillation frequency and magnitude changed significantly from baseline at 5% injury. Oscillations were not present in simulations with greater than 80% injury, marked by the vertical dashed line. (E) Random neurodegeneration did not significantly impact oscillation FWHM until 60% injury.
