# Supplementary material for: Neurodegeneration exposes firing rate dependent effects on oscillation dynamics in computational neural networks
Source: PLoS One. 2020 Sep 23;15(9):e0234749. doi: 10.1371/journal.pone.0234749 (PMC7510994; doi:10.1371/journal.pone.0234749)
Supplement: S2 Fig — (A) Networks consisting of RS or IB excitatory neurons had reduced average network firing rate compared to uninjured baseline networks at 25% damage. Networks with CH excitatory neurons responded less to injury and, thus, sustained activity near baseline levels. (B) At 50% damage, networks with RS or IB excitatory neurons showed larger decreases in firing rate. Networks with chattering neurons remained resilient. Inhibitory neuron subtype did not significantly affect the network response to damage. (PDF) [file pone.0234749.s002.pdf]

S2 Fig Supplementary Information

Although our primary goal was to model a generic cortical circuit, there are, in fact, many different types of excitatory and inhibitory neurons. These would be especially relevant in a model of a specific region of the brain. As we work to develop these more precise circuits, we were interested in exploring the diversity of neuronal spiking behaviors and how the effects of damage might differ in a circuit with different types of neurons. To do so, we examined the interactions between excitatory and inhibitory neurons with different spiking behaviors.

In networks of 1000 neurons, we constructed networks with 80% excitatory and 20% inhibitory neuron but varied the specific excitatory and inhibitory cell type. We considered the following three excitatory neuron types: regular spiking (RS), intrinsically bursting (IB), and chattering (CH). We used the following three inhibitory neuron types: fast spiking (FS), regular spiking (RS), and intrinsically bursting (IB). Following our normal procedure, we ran with plasticity to settle the network and subsequently implemented random damage at 25 and 50%. We then compared the acute change in firing rate after injury and found that the excitatory composition of the network is primarily responsible for altered activity post-injury.

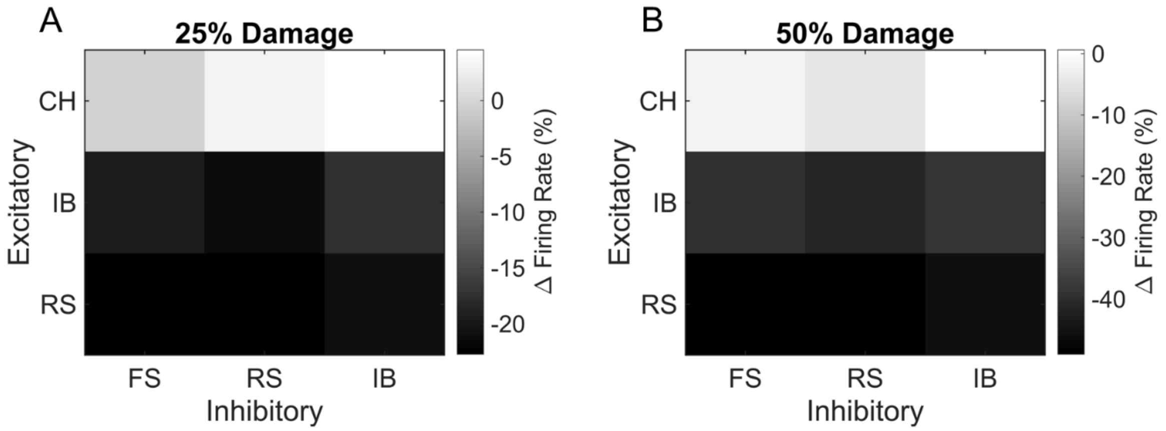

S2 Fig. Electrophysiology and spiking behaviors of neurons affect network response to damage. (A) Networks consisting of RS or IB excitatory neurons had reduced average network firing rate compared to uninjured baseline networks at 25% damage. Networks with CH excitatory neurons responded less to injury and, thus, sustained activity. (B) At 50% damage, networks with RS or IB excitatory neurons showed larger decreases in firing rate. Networks with chattering neurons remained resilient. Inhibitory neuron subtype did not significantly affect the network response to damage.

Notably, we find that the subtype of inhibitory neuron does not play a significant role in the firing rate of the overall network after damage with any excitatory network composition or damage level ($p<0.05$; ANOVA with Bonferroni correction for multiple comparisons, $p<0.05$). It is possible and likely that these

inhibitory subtypes do affect higher level network function (e.g., oscillations, synchronization, etc.). Their functional significance in the circuit may arise through their influence on spike timing beyond straightforward activity rate.

Compared to inhibitory neuron type, we find that the excitatory neuron spiking behavior has a much larger effect on the network firing rate after injury. In networks with intrinsically bursting or regular spiking neurons, decreases in firing rate were consistent with our testing in the primary manuscript (IB: -19.4% ± 3.24 and -39.8% ± 2.62 at 25 and 50% damage, respectively. RS: -22.0% ± 3.16 and -47.6% ± 3.18 at 25 and 50% damage). Interestingly, networks of chattering excitatory neurons maintained their firing rate at both levels of injury (Ch: -2.46% ± 4.46 and -2.02% ± 5.09 at 25 and 50%). The resilience of these chattering neuron networks may be due to the inherent patterned activity of these excitatory neurons. Their intrinsic electrophysiological properties are enough to maintain firing, even with fewer synaptic inputs.

While these simulations provide some insight into how the effects of damage vary depending on the neuron composition of a neural circuit, the potential effects of damage on different electrophysiological behaviors of neurons within small circuits, further research into the interaction of these neuron subtypes can improve our knowledge on how real circuits would be affected by degeneration.
